# Strengthening bonds of Friendship between Pakistan and China through scientific publishing

**DOI:** 10.12669/pjms.37.6-WIT.5037

**Published:** 2021

**Authors:** Shaukat Ali Jawaid

Peoples Republic of China is the best friend of Pakistan who has always supported us on various international forums. Not only that China has maintained close and cordial relationship with Pakistan in different fields all these years. More recently, it has made huge investment through CPEC project which will play a vital role in economic development of Pakistan. Over the years, China has also made important progress and has emerged as one of the fastest developing economies and a world economic power and it is increasing its sphere of influence all over the world helping many countries in different fields. At present after United States of America, China is the biggest contributor to the world medical literature if we look at the number of papers published from China.

We in Pakistan Journal of Medical Sciences have been regularly receiving scientific papers from authors from China for the last many years and the number of annual submissions have been increasing almost every year. If in 2011 the number of submissions from China were sixty four, it increased to over five hundred during the years 2014, 2015 and 2016. However, we could accept only a few for publication after peer review which were either of interest to our readers in this part of the world and the authors were willing to revise and resubmit after peer review responding to the reviewer's comments and suggestions.^1^ Fig-1.

It was in March 2021 that we received a communication from Dr. Shaofei Wu from Wuhan Institute of Technology with a proposal to publish a special issue highlighting the research activity of their institution in the field of medical imaging. They wanted us to showcase their research by publishing a special issue. In the past we have published a special issue highlighting the research being carried at Isfahan University of Medical Sciences in March 2013.^2^ More recently we published a special issue highlighting the research activity undertaken by Indus Health Network in November 2019.^3^ Yet another special issue was devoted to PRIME Guidelines by Baqai Institute of Diabetes and Endocrinology which was published in August 2016.^4^ Hence keeping in view the cordial relations that we have with China, we decided to accept that proposal with the following terms and conditions for the editorial contents:

1. The institution will select one or two Guest Editors and they will be overall responsible for the contents of this issue.
2. The authors will follow the instructions for authors on our website while preparing the manuscripts and they will be particularly mindful about the length of the manuscript.
3. It will be an Only Online issue and no print copy will be available hence no print copy will be supplied.
4. The Guest Editors will ensure that every manuscript being contributed to this issue will go through proper peer review system. They will submit these peer review reports along with the submission.
5. Every manuscript will be accompanied by Letter of Undertaking signed by all the listed authors confirming the exclusive submission to Pakistan Journal of Medical Sciences.
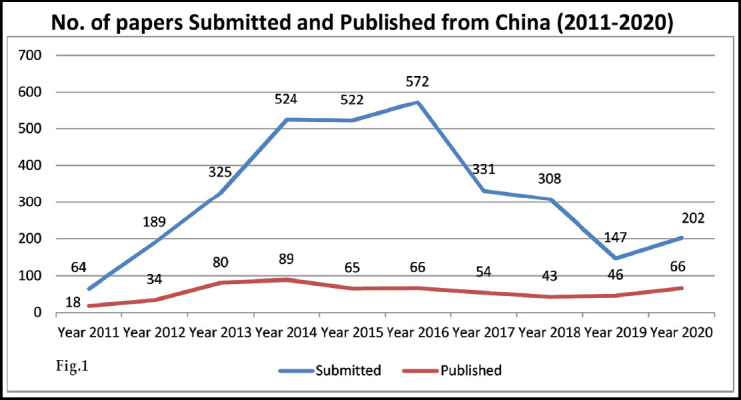
6. The authors will keep in mind the authorship criteria by International Committee of Medical Journals Editors (ICMJE) as we do not wish to promote Gift authorship a menace which is very common all over the words these days, China being no exception.
7. It was also made clear that in most cases we allow just four authors just to discourage gift authorship but in some cases based on the study, one can allow more authors if all those listed as authors deserve authorship.
8. Every manuscript will also be accompanied by approval from the Ethics Committee/Institutional Review Board with Reference Number, Date and stamp of the institution of the Ethics Committee.
9. We will do the copy editing and reserve the right to reject any manuscript if we are not satisfied with the peer review report or think it is beyond the scope of our journal.
10. The authors might have to revise their manuscripts one again after editing if we feel such a need and the authors will be given guidelines in this respect.
11. All papers received will be checked for plagiarism using iThenticate software and if similarity index score is more than 18%, such papers will be rejected.
12. After the papers have been submitted, we will check them for plagiarism, format them after editing and then upload the edited version on our website before further processing.
13. PDF files of all the papers will be sent to the organizers to share with the authors for proofreading before they are finally published.
14. Once the issue has been finalized and published online, we will make arrangements for preparing the XML files for submission to PubMed Central and the issue will be uploaded as soon as possible so that it is visible on Medline, PubMed, PubMed Central as well.
15. We had also made it clear that we will not accommodate more than thirty manuscripts in this special issue and they will be selected by the Guest Editors from the large number of papers available.

Once an agreement was reached, we received thirty-two papers for this issue. Initial review of the submissions disclosed that most of the authors had failed to carefully read and follow the instructions for authors as the papers were too lengthy with large number of figures, illustrations and tables in each manuscript. Our observations were conveyed to the Guest Editors and we expressed our inability to proceed further unless the authors revise the manuscript in the light of the instructions on our website. They were also given the option that if they cannot do that and were not feeling comfortable, we can abandon this project but we were not prepared to compromise on the quality. The message came that they were willing to revise the papers once again. At this, they were asked to first revise just two papers and send it to us for perusal. If we felt satisfied, only then they should proceed further and submit the remaining manuscripts. After few days we received the two revised papers which we found in order hence they were asked to proceed with further submissions.

Thirty-one papers were found in order while one papers was rejected because it was based on the data already covered in another manuscript and it was almost a duplicate submission. During further editing, a number of paragraphs needed to be rewritten, some of the sentences had to be rephrased to ensure that the message was conveyed effectively. In some cases, even we were at a loss as to what the authors wanted to convey, hence could not rewrite and rephrase them. As such these paragraphs were shared with the Guest Editors asking them to request the authors to rewrite them so that they can be replaced in their manuscripts. All this went on smoothly and the guest editors were kind enough to persuade the respective authors to respond immediately.

This special issue now contains thirty-one manuscripts highlighting different aspects of medical imaging, X-ray, Ultrasound, CT Scan, MRI, computer aided diagnosis, latest developments in medical imaging. As pointed out by the Guest Editors, it focuses on human physiology and physiological dysfunction, medical imaging and physical analysis methods, medical functional imaging, medical image processing methods, computational fluid dynamics, medical engineering materials besides medical image signal processing and physiological signal monitoring. All this are extremely helpful in non-invasive diagnosis and treatment methods. They are also helpful in early diagnosis and prevention of diseases.

We sincerely hope that our readers will find this issue highly informative and it will help them in their professional capacity building in their respective areas of expertise. At the same time, it will not only help improve patient care but also go a long way in further cementing our brotherly relations with Peoples Republic of China. We will continue to strive for greater cooperation and collaboration between the healthcare professionals of both the countries, the academicians and researchers in particular which will be to our mutual benefit.
